# Ionizing Radiation-Induced GDF15 Promotes Angiogenesis in Human Glioblastoma Models by Promoting VEGFA Expression Through p-MAPK1/SP1 Signaling

**DOI:** 10.3389/fonc.2022.801230

**Published:** 2022-02-25

**Authors:** Hyejin Park, Ki-Seok Nam, Hae-June Lee, Kwang Seok Kim

**Affiliations:** ^1^ Division of Radiation Biomedical Research, Korea Institute of Radiological and Medical Sciences, Seoul, South Korea; ^2^ School of Radiological and Medico-Oncological Sciences, University of Science and Technology, Daejeon, South Korea

**Keywords:** GDF15, glioblastoma, endothelial cells, radiotherapy, angiogenesis

## Abstract

Glioblastoma multiforme (GBM), the most aggressive cancer type that has a poor prognosis, is characterized by enhanced and aberrant angiogenesis. In addition to surgical resection and chemotherapy, radiotherapy is commonly used to treat GBM. However, radiation-induced angiogenesis in GBM remains unexplored. This study examined the role of radiation-induced growth/differentiation factor-15 (GDF15) in regulating tumor angiogenesis by promoting intercellular cross-talk between brain endothelial cells (ECs) and glioblastoma cells. Radiation promoted GDF15 secretion from human brain microvascular endothelial cells (HBMVECs). Subsequently, GDF15 activated the transcriptional promoter *VEGFA* in the human glioblastoma cell line U373 through p-MAPK1/SP1 signaling. Upregulation of vascular endothelial growth factor (VEGF) expression in U373 cells resulted in the activation of angiogenic activity in HBMVECs *via* KDR phosphorylation. Wound healing, tube formation, and invasion assay results revealed that the conditioned medium of recombinant human GDF15 (rhGDF15)-stimulated U373 cell cultures promoted the angiogenic activity of HBMVECs. In the HBMVEC-U373 cell co-culture, *GDF15* knockdown mitigated radiation-induced VEGFA upregulation in U373 cells and enhanced angiogenic activity of HBMVECs. Moreover, injecting rhGDF15-stimulated U373 cells into orthotopic brain tumors in mice promoted angiogenesis in the tumors. Thus, radiation-induced GDF15 is essential for the cross-talk between ECs and GBM cells and promotes angiogenesis. These findings indicate that GDF15 is a putative therapeutic target for patients with GBM undergoing radio-chemotherapy.

## Introduction

Glioblastoma multiforme (GBM), which is the most common malignant brain tumor in adults, is characterized by enhanced vascularization and a complex vascular phenotype (1, 2). Angiogenesis plays an important role in the progression of glioma (3, 4). To maintain proliferation, cancer cells secrete multiple factors that promote the formation of new blood vessels, which supply oxygen and nutrition to them (5, 6). Therefore, anti-angiogenesis drugs are commonly used to treat GBM (2, 4, 7). Additionally, GBM is treated using radiotherapy to inhibit angiogenesis. However, angiogenesis is reactivated within a short duration of treatment cessation (8, 9).

Vascular endothelial growth factor (VEGF) is an essential paracrine factor involved in maintaining vascular homeostasis and mediating pathological angiogenesis. Generally, endothelial cells (ECs) produce VEGF only in response to radiation doses of > 10 Gy (10). However, radiation activates hypoxia-inducible factor-1 (HIF1) in tumors and promotes the survival and proliferation of ECs through the induction of VEGF expression (11).

Growth/differentiation factor-15 (GDF15), a 34 kDa secretory protein belonging to the transforming growth factor-beta (TGFβ) superfamily, is involved in the development and regulation of cardiac vascular diseases, as well as in hormone responses that maintain systemic homeostasis (12, 13). Previous studies have reported that upregulated GDF15 expression protects against ischemia/reperfusion injury (also called acute EC apoptosis) during heart transplantation (14). In ECs, irradiation upregulates GDF15 levels, which leads to enhanced oxidative stress and cellular senescence (15). Although GDF15 has been well characterized, its role in cancer progression remains unclear. Inhibition of the p38/MEK signaling pathway mitigates the GDF15 overexpression-induced invasion and proliferation of ovarian cancer cells (16). Osteocyte-derived GDF15 enhances prostate cancer cell proliferation and invasion by promoting the interaction between osteocytes and prostate cancer cells (17). In non-small cell lung cancer cells, GDF15 arrests the cell cycle at the G0/G1 phase, leading to cellular apoptosis (18). However, the role of GDF15 in cancer progression has not been elucidated as its expression levels vary among patients and cancer types (19, 20).

In this study, we hypothesized that irradiation-induced GDF15 promotes angiogenesis in glioblastoma by functioning as a cytokine in the tumor microenvironment. This study aimed to examine the role of GDF15 in the cross-talk between a human GBM cell line (U373) and human brain microvascular endothelial cells (HBMVECs; representative ECs). In addition, the mechanisms underlying GDF15-mediated regulation of angiogenesis in GBM were examined.

## Materials and Methods

### Materials

Recombinant human GDF15 (rhGDF15) was purchased from PeproTech (#120-28C, Cranbury, NJ, USA). U0126, a MEK/MAPK1 specific inhibitor, was obtained from Promega Co. (#V112A, Madison, WI, USA). Anti-VEGF antibody (#MAB293) was obtained from R&D Systems (Abingdon, UK).

### Cell Culture

HBMVECs purchased from iXCells Biotechnologies (CA, USA) were cultured in EGM™-2 Endothelial Cell Growth Medium-2 BulletKit™ (#CC-3162, Lonza, MD, USA) at 37°C and 5% CO_2_ in a humidified incubator. Cells passaged for 5–7 times were used for the experiments. For conducting ionizing radiation (IR) experiments, the cells were irradiated with γ-rays at a dose of 3.5 Gy/min using a ^137^Cs γ-ray source (Atomic Energy of Canada, Ltd., ON, Canada). U373 cells were cultured in Dulbecco’s modified Eagle’s medium supplemented with 10% fetal bovine serum (FBS; #35-015-CV, Corning, NY, USA) and 1% penicillin-streptomycin (#15240-062, Thermo Fisher Scientific, UK). To analyze cell growth, U373 cells were cultured in 12-well plates (5 × 10^4^ cells/well) for 3 days. Cells cultured under different serum concentrations (2% and 10%) were counted daily.

### Wound Healing Assay

U373 cells (3 × 10^5^ cells/well) and HBMVECs (1.5 × 10^5^ cells/well) were seeded in 12-well cell culture plates and incubated overnight. A scratch was introduced in the monolayer using a pipette tip. The culture medium was replaced to remove cell debris. After washing, three or more images were taken, and then the cells were cultivated for 24 h with rhGDF15 (#120-28C, PeproTech, NJ, USA). The next day, images of the same location as that of the previous day were taken. Using ImageJ line tool, a line was drawn along the area newly filled by proliferated cells. The areas calculated by ImageJ were averaged (three or more images per group) and normalized to the control group.

### Tube Formation Assay

Individual wells of a 24-well cell culture plate were coated with 250 µL of Matrigel (#354234, Matrigel® Basement Membrane Matrix, LDEV-free, Corning, NY, USA) for 1 h before cell seeding. HBMVECs (1 × 10^5^ cells/well) were seeded in the coated wells and cultured for 12 h. The tube shapes were analyzed using ImageJ software (ver.1.52a with angiogenesis analyzer plugin).

### Co-Culture of HBMVECs and U373 Cells

HBMVECs and U373 cells were cultured in a co-culture system. A 12-mm Transwell with a polycarbonate membrane insert (pore size: 0.4 μm; #CLS3413, Corning, NY, USA) was used. In the upper well, U373 cells (5 × 10^4^ cells) were seeded in culture medium supplemented with 2% FBS. HBMVECs (1.5 × 10^5^ cells) were seeded in the bottom well containing EC culture medium supplemented with 2% FBS. The monolayer of HBMVECs was scratched with a tip to introduce a wound. The culture medium was replaced with a fresh culture medium. Next, the cells were irradiated with 8 Gy IR.

### Quantitative Reverse Transcription Polymerase Chain Reaction (qRT-PCR)

Total RNA was extracted using QIAzol Lysis Reagent (#79306, Qiagen, Hilden, Germany), following the manufacturer’s instructions. The isolated RNA was reverse-transcribed into cDNA using amfiRivert cDNA Synthesis Platinum Master Mix (#R5600, GenDEPOT, Barker, TX, USA). qRT-PCR analysis was performed using the SYBR Green 2× Master Mix kit (#18303, Mbiotech, Inc., Gyeonggi, Korea) and a CFX96 Touch™ Real-Time PCR detection system (Bio-Rad, Hercules, CA, USA). The PCR conditions were as follows: initial denaturation at 95°C for 2 min, followed by 45 cycles at 95°C for 10 s, 60°C for 5 s, and 72°C for 12 s. The results were analyzed using CFX Manager™ software, version 2.1. The expression of target genes was normalized to that of 18S RNA. The following primers were used: *GDF15*, 5′-GACATCACTAGGCCCCTGA-3′ (forward) and 5′-CCCGTAAGCGCAGTTCC-3′ (reverse); *VEGFA*, 5′-TCAGTTCGAGGAAAGGGAAA-3′ (forward) and 5′-GAGGCTCCAGGGCATTAGAC-3′ (reverse); *kinase insert domain receptor* (*KDR*), 5′-CGGTCAACAAAGTCGGGAGA-3′ (forward) and 5′-CAGTGCACCACAAAGACACG-3′ (reverse); and 18S RNA, 5′-GGCCCTGTAATTGGAATGAGTC-3′ (forward) and 5′-CCACGATCCAACTACGAGCTT-3′ (reverse).

### Western Blotting

Cells were lysed in ice-cold protein extraction solution (#EBA-1049, ELPIS-BIOTECH, Daejeon, Korea). Equal amounts of protein (40 µg) were subjected to sodium dodecyl sulfate (SDS)-polyacrylamide gel electrophoresis. The resolved proteins were transferred to a nitrocellulose membrane. The membrane was probed with the following primary antibodies: anti-GDF15 (1:1000, #SC66904, Santa Cruz Biotechnology, Inc., TX, USA), anti-MAPK1 (1:1000, #9102, Cell Signaling Technology, Inc., MA, USA), anti-p-MAPK1 (1:1000, #9101, Cell Signaling Technology, Inc.), anti-SP1 (1:1000, #SC14027, Santa Cruz, CA, USA), anti-VEGF (1:1000, #SC1836, Santa Cruz Biotechnology, Inc.), anti-KDR (1:1000, #9698 Cell Signaling Technology, Inc.), anti-p-KDR (1:500, #3817, Cell Signaling Technology, Inc.), and anti-ACTB (1:3000 #SC47778, Santa Cruz Biotechnology, Inc.) antibodies. ACTB was used as the loading control. Immunoreactive signals were developed using enhanced chemiluminescence.

### Promoter Assay

Variant VEGF constructs were used as luciferase-based reporters for the *VEGFA* promoter region (pGL4.10-VEGFprom −1000 to −1, −950 to −700, −1000 to −500, and −500 to −1 bp). The following VEGFA constructs were purchased from Addgene (MA, USA): pGL4.10-VEGFprom −1000 to −1 (plasmid #66128); pGL4.10-VEGFprom −950 to −700 (plasmid #66133); pGL4.10-VEGFprom −1000 to −500 (plasmid #66129); and pGL4.10-VEGFprom −500 to −1 (plasmid #66130). The constructs were transfected into 293T cells, and the cells were treated with 50 ng/mL of rhGDF15. The cells were lysed using the Nano-Glo® Dual-Luciferase® Reporter Assay System (Promega, WI, USA). The luminescence intensity in the lysate was measured using a GloMax® Discover System.

### Chromatin Immunoprecipitation (ChIP)

U373 cells were treated with 10 µM U0126 for 1 h, followed by treatment with 100 ng/mL rhGDF15. The cells were harvested, lysed in SDS with 50 mM Tris-HCl (pH 8.1) and 1 mM ethylenediaminetetraacetic acid (EDTA), and sonicated for 1 h at 4°C. The supernatant was evenly split and incubated with 2 μg of anti-SP1 antibody overnight at 4°C with rotation. The reaction mixture was incubated with protein A beads (60 μL/reaction) for 1 h at 4°C. The beads were then washed thrice. The eluted supernatants and input DNA samples were incubated at 65°C for 4 h to allow de-crosslinking. The DNA was then precipitated with ethanol and treated with proteinase K for 30 min at 37°C. The samples were extracted with phenol/chloroform, precipitated with ethanol, and resuspended in 20 μL of distilled water. The promoter-binding activity was measured using qRT-PCR analysis with specific promoter primers (forward, 5′-GGGTAGCTCGGAGGTCGT-3′; reverse, 5′-GGGAATGGCAAGCAAAAA-3′).

### Immunocytochemistry

HBMVECs were seeded in 12-well dishes on coverslips. The cells were fixed in 3.7% formaldehyde/phosphate-buffered saline (PBS) for 10 min at room temperature (RT) and permeabilized with ice-cold 0.5% Triton X-100/PBS for 10 min. The permeabilized cells were blocked with 0.5% bovine serum albumin (BSA)/PBS for 1 h at RT and incubated with anti-GDF15 antibodies overnight at 4°C. Next, the cells were treated with goat anti-rabbit IgG (H+L) Alexa Fluor^®^ 488 (#A27034, Invitrogen Inc., Carlsbad, CA, USA) for 1 h. The coverslips were mounted on glass slides with Fluoromount-G™ Mounting Medium (#0100-01, Southern Biotech, AL, USA).

### Enzyme-Linked Immunosorbent Assay (ELISA)

To analyze the concentration of soluble proteins (GDF15 and VEGFA), the cultured media were passed through 0.45-μm filter membranes and concentrated using Vivaspin^®^ 20 centrifugal concentrators (Sartorius, Göttingen, Germany). ELISA was performed using commercial kits (GDF15, #DGD150; VEGF, #DVE00; R&D Systems, Inc., MN, USA), following the manufacturer’s instructions. Briefly, the standard was prepared by serially diluting recombinant proteins. The samples were then loaded into the plates with anti-GDF15 or anti-VEGFA antibodies for 2 h at RT. Next, the plate wells were incubated with the substrate solution for 30 min, followed by incubation with a stop solution. The absorbance of the reaction mixture was measured at 450 nm.

### Small Interfering RNA (siRNA) Transfection

To knockdown *GDF15*, HBMVECs were transfected with *GDF15* siRNAs (siGDF15; 50 nM, ON-TARGETplus siRNAs, Dharmacon Inc., CO, USA) or control siRNA (siCon) using Lipofectamine 2000 (Invitrogen Inc., CA, USA), following the manufacturers’ instructions. Briefly, 1 × 10^6^ cells were seeded in a 60 mm culture dish and cultured overnight. Lipofectamine was incubated with 50 ng/mL siRNA for 20 min. The cells were washed twice with PBS, and the culture medium was replaced with EBM™-2 Endothelial Cell Growth Basal Medium (#CC-3156, Lonza). Next, the cells were incubated with the siRNA/Lipofectamine mixture for 6 h. The medium was then replaced with EGM™-2 Endothelial Cell Growth Medium-2 BulletKit™ (#CC-3162, Lonza). The cells were harvested 48 h after transfection.

### Orthotopic Brain Tumor Model

All animal experiments were approved by the Institutional Animal Care and Use Committee of the Korea Institute of Radiological and Medical Sciences (Approval no. KIRAMS2018-0079). Athymic BALB/c nu/nu mice were purchased from Orient Bio Inc. (Seoul, Korea). The mice were provided standard food and tap water under specific pathogen-free conditions. Prior to injecting the U373 cells into the mouse brain, the cells were treated with GDF15 by adding rhGDF15 to the culture medium for 14 days. The culture medium was changed every three days with fresh rhGDF15-containing medium. For injecting U373 cells (1 × 10^5^/3 µL), the head of each mouse was fixed on a stereotactic device. The cells were injected into the left frontal cortex using a microinjector. On day 10 post-injection, mouse brain samples were harvested, fixed in 4% paraformaldehyde solution, embedded in paraffin, and cut into 5 µm-thick sections using a microtome (Leica, Nussloch, Germany).

### Tumor Size Evaluation

The brain tissue was coronally sectioned to a thickness of 5 µm using a microtome (Leica, Nussloch, Germany). The sections were hydrated and stained with hematoxylin and eosin (H-E). The area of the tumor with the greatest longitudinal diameter and transverse diameter was measured. The U373 cell-derived tumor was manually traced along the tumor margin, and the area was measured using ImageJ.

### Immunofluorescence Analysis of Brain Tumor

The embedded brain tissues were sectioned to a thickness of 5 µm using a microtome (Leica, Nussloch, Germany). The sections were mounted on coated slides (#631-1349, Adhesion slides, Menzel Gläser, Polysine^®^, Thermo Fisher Scientific, UK), hydrated, and boiled in citrate buffer (pH 6.0) for antigen retrieval. After washing twice with 0.1% Triton X-100/PBS for 5 min, the sections were blocked with 1.5% normal horse serum for 1 h at RT to inhibit non-specific signals. The sections were subsequently incubated overnight at 4°C with anti-VEGFA (1:200, #SC1836, Santa Cruz Biotechnology), anti-CD31 (1:200, # AF3628, R&D Systems, MN, USA), anti-p-MAPK1 (#9102, Cell Signaling Technology, Inc), and anti-SP-1 antibodies, followed by incubation with the appropriate secondary antibodies for 1 h at RT. The slides were observed using an Olympus BX53 fluorescence microscope with DP73 digital camera (Olympus, Tokyo, Japan). Three fields were arbitrarily photographed for each tumor, and the average value of the three fields was used as a representative value for one tumor. The fluorescence intensity was analyzed by Image J using the following formula: Corrected Total Cell Fluorescence (CTCF) = Integrated Density − (Area of selected cell × Mean fluorescence of background readings).

### Statistical Analysis

All data are presented as mean ± standard deviation. The means between two groups were compared using Student’s *t*-tests, whereas those among more than two groups were compared using one-way or two-way analysis of variance. Differences were considered significant at *p* < 0.05. All statistical analyses were performed using GraphPad Prism Software version 8.3 (GraphPad Software, La Jolla, CA, USA).

## Results

### IR Upregulates GDF15 and Promotes Its Secretion From HBMVECs

**Figure 1:**
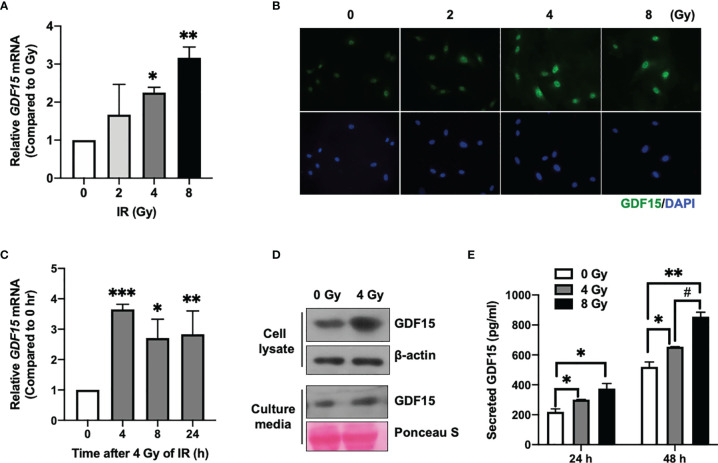
Effect of ionizing radiation (IR) on GDF15 expression in human brain microvascular endothelial cells (HBMVECs). **(A)** Effect of different doses of IR on *GDF15* mRNA expression in HBMVECs. Cultured cells were harvested 24 h post-IR exposure, and *GDF15* mRNA levels were analyzed using quantitative reverse transcription polymerase chain reaction (qRT-PCR). **(B)** Effect of different doses of IR (0 – 8 Gy) on GDF15 protein levels in HBMVECs. GDF15 protein expression was detected by immunofluorescence analysis using anti-GDF15 antibody (green). Nuclei were stained with 4′,6-diamidino-2-phenylindole (DAPI) (blue) (Magnification: 400×); **(C)** Time course of *GDF15* mRNA expression after irradiation with 4 Gy of IR. *GDF15* mRNA levels measured using qRT-PCR at the indicated time points. **(D)** Immunoblotting analysis of GDF15 in the cell lysate and culture medium reveals that IR upregulated GDF15 protein levels. Cells and culture media were harvested at 24 h post-IR exposure. **(E)** IR promotes the secretion of GDF15. The secretion of GDF15 into the culture medium was measured using a human GDF15 enzyme-linked immunosorbent assay kit at 24 and 48 h post-irradiation with 4 or 8 Gy of IR. Data are presented as the mean ± standard deviation of three experiments. *p < 0.05, **p < 0.01, and ***p < 0.001 compared with the control. ^##^ *p* < 0.05 compared with 4 Gy.

The effect of different doses of IR (2, 4, and 8 Gy) on *GDF15* mRNA expression in HBMVECs was examined 24 h post-irradiation. *GDF15* mRNA levels in cells irradiated with IR at doses of ≥ 4 Gy were significantly upregulated compared to those in the nonirradiated cells (**Figure 1A**). Consistent with its effect on *GDF15* mRNA levels, IR increased intracellular GDF15 immunofluorescence signals (**Figure 1B**). qRT-PCR analysis revealed that the upregulation of *GDF15* mRNA levels was significant 4 h post-irradiation onward (**Figure 1C**). Immunoblotting analysis revealed that GDF15 protein levels were upregulated in cell lysates and cell culture media (secreted GDF15) 24 h post-irradiation (**Figure 1D**). Consistently, ELISA results revealed that IR promoted GDF15 secretion into the culture medium of HBMVECs (**Figure 1E**). These results indicate that IR upregulated endogenous/cytosolic GDF15 expression and secretion in HBMVECs. The cytosol and extracellular milieu are important components of the tumor microenvironment.

### GDF15 Promotes VEGFA Expression in U373 Cells But Not U373 Cell Invasion or Proliferation

**Figure 2:**
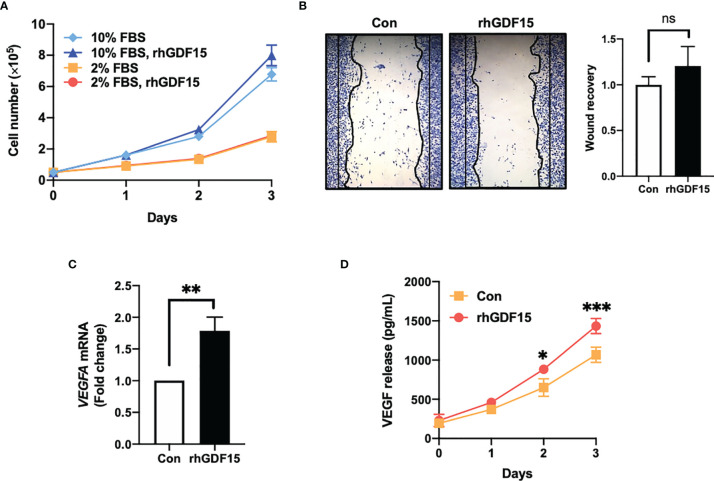
GDF15 promotes VEGFA expression in U373 glioblastoma cells but not proliferation and migration of U373 cells. **(A)** U373 cells were treated with 100 ng/mL rhGDF15 protein for 3 days in a medium with different serum concentrations (2% or 10% fetal bovine serum) and the cells were counted daily. **(B)** The cells were seeded on 12-well plates (3 × 10^5^ cells/well) and cultured for 1 day. The monolayer was scratched and incubated for 24 h in a medium containing 2% serum. Subsequently, the cells were stained with a fixation solution containing trypan blue. **(C)** The *VEGFA* mRNA levels in rhGDF15-stimulated U373 cells were measured using quantitative reverse transcription polymerase chain reaction. **(D)** Soluble VEGF was quantified in the enriched culture medium containing 2% serum using enzyme-linked immunosorbent assay from days 1–3. Data are presented as the mean ± standard deviation of three experiments. *p < 0.05, **p < 0.01, and ***p < 0.001 compared with control group. ns, no significance.

To investigate the role of GDF15 in glioblastoma, the effect of GDF15 on the *in vitro* proliferation and invasion of U373 cells was examined. GDF15 is reported to promote cancer cell growth (16, 17). Hence, the effect of rhGDF15 on the proliferation and migration of U373 cells was examined in this study. U373 cells were cultured for up to 3 days in the presence of rhGDF15 (in the medium supplemented with 10% serum or a serum substitute); rhGDF15 did not affect the proliferation rate of U373 cells (**Figure 2A**). The results of the wound healing assay revealed that rhGDF15 increased the migration of U373 cells, although the change was not statistically significant (*p* = 0.4248; **Figure 2B**).

GDF15, which has a structure similar to that of TGFβ, is a member of the TGFβ superfamily. The TGFβ superfamily members can stimulate cytokine-inducible factors, including HIF1a, C-X-C motif chemokine ligands (CXCLs), and VEGF, in cancer cells (4). Thus, the effect of GDF15 on VEGFA expression in U373 cells was examined. The mRNA and secretory levels of VEGFA in rhGDF15-treated U373 cells were examined. Interestingly, *VEGFA* mRNA levels in the rhGDF15-stimulated U373 cells were upregulated by 1.78-fold compared to those in the control U373 cells (*p* = 0.0032; **Figure 2C**). Meanwhile, soluble VEGFA levels in the culture medium of rhGDF15-treated cells were significantly higher than those in the culture medium of control cells (*p* = 0.0131 and *p* = 0.0008 at days 2 and 3 post-rhGDF15 treatment, respectively; **Figure 2D**).

We also examined the effect of GDF15 on HBMVECs. We observed that GDF15 increased the viability, wound recovery ability, and tube formation ability of these cells; however, these observations were not statistically significant. Additionally, we observed no significant effect on pMAPK by western blotting (**Supplementary Figure S1**).

### GDF15 Stimulates *VEGFA* Promoter Activity in U373 Cells *via* the p-MAPK1/SP-1 Pathway

**Figure 3:**
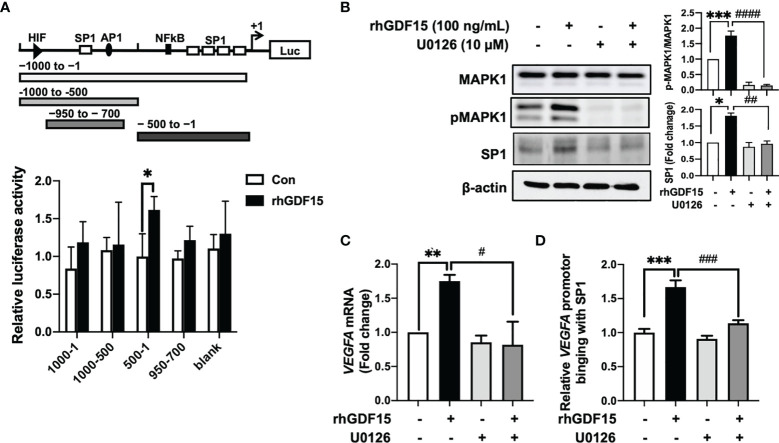
GDF15 upregulates *VEGFA* transcription by promoting the binding of SP1 to its promoter. **(A)** 293T cells were transfected with a luciferase construct containing different *VEGFA* promotor regions or control vectors and treated with recombinant human GDF15 (rhGDF15). Data are presented as the mean ± standard deviation of three experiments (*p < 0.05). **(B)** For immunoblotting analysis of MAPK1, p-MAPK1, and SP1 in U373 cells, the cells were incubated for 1 h in the presence or absence of a MAPK1 inhibitor (U0126) and treated with rhGDF15 for 6 h. **(C)** Quantitative reverse transcription polymerase chain reaction (qRT-PCR) analysis of *VEGFA* mRNA expression in U373 cells treated with rhGDF15 and/or U0126. qRT-PCR analysis was performed using samples extracted 12 h post-rhGDF15 treatment. For inhibition studies, U373 cells were incubated with 10 µM U0126 for 1 h before treatment with 100 ng/mL rhGDF15. **(D)** Cells were incubated with or without rhGDF15 and U0126 and subjected to chromatin immunoprecipitation (ChIP) assay using anti-SP1 or IgG isotype control antibodies. Bar graphs represent the qRT-PCR results for immunoprecipitated *VEGFA* promoter. Data are presented as the mean ± standard error (**p < 0.01 and ***p < 0.001 compared with control group; ^#^ *p* < 0.05, ^##^ *p* < 0.01, ^###^ *p* < 0.001 and ^####^ *p* < 0.0001 compared with rhGDF15-treated group.

To investigate the mechanism underlying GDF15-mediated regulation of VEGFA expression, the effects of rhGDF15 on the luciferase activity of various *VEGFA* promoter-reporter constructs were examined. GDF15 activated the GC-rich *VEGFA* promoter containing an SP1-binding site (from −500 to −1) (**Figure 3A**). Transcription factors, such as NF-κB and STAT3 can bind to the GC-rich *VEGFA* promoter (21). However, GDF15 did not activate NF-κB and STAT3 expression in U373 cells (**Supplementary Figure S2**). This indicated that VEGFA is induced by the MAPK1 signaling pathway through the transcription factor SP1 (22, 23). In order to inactivate MAPK1, U373 cells were treated with U0126 1 h before the addition of recombinant hGDF15. The cells were incubated for 6 h and then harvested for western blotting. rhGDF15 treatment increased MAPK1 phosphorylation and SP1 levels in U373 cells (**Figure 3B**). U0126 suppressed SP1 expression and MAPK1 phosphorylation in rhGDF15-stimulated and control U373 cells (**Figure 3B**). To confirm the role of GDF15-induced VEGFA expression, *VEFGA* expression was examined by qRT-PCR analysis 12 h after incubation with rhGDF15 and/or U0126. qRT-PCR analysis revealed that U0126 mitigated the GDF15-induced upregulation of *VEGFA* mRNA levels (**Figure 3C**). Next, the effect of GDF15 on the direct binding of SP1 to the *VEGFA* promoter was examined using a ChIP assay to confirm the GDF15-mediated regulation of VEGFA expression through MAPK1/SP1 signaling. The results of a series of ChIP assays using anti-SP1 antibodies revealed that GDF15 promoted the binding of SP1 to the *VEGFA* promoter and that U0126 mitigated the GDF15-induced SP1 promotor binding (**Figure 3D**). These data suggest that GDF15 activates transcription of the *VEGFA* promoter through p-MAPK1/SP1 signaling.

### GDF15 Modulates the Interaction Between ECs and Glioblastoma Cells

**Figure 4:**
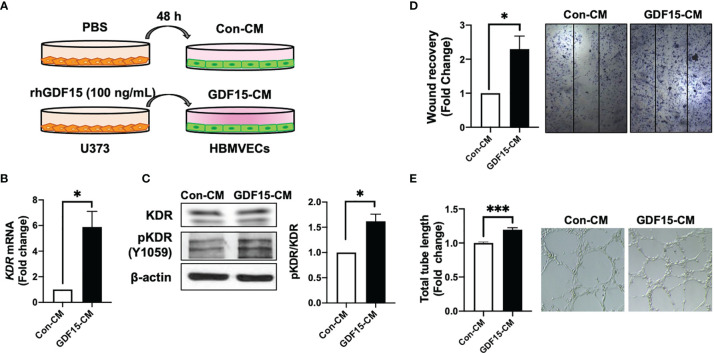
GDF15-induced VEGFA activates angiogenesis. **(A)** Schematic representing human brain microvascular endothelial cells (HBMVECs) cultured in control conditioned medium (Con-CM; conditioned medium from U373 cells) or GDF15-CM (conditioned medium from rhGDF15-stimulated U373 cells); **(B)** *KDR* mRNA expression in HBMVECs was measured using quantitative reverse transcription polymerase chain reaction (qRT-PCR). **(C)** Phosphorylation of KDR was examined using western blotting (β-actin as used as loading control); **(D)** Wound healing assay results. The day before the experiment, HBMVECs were seeded in 12-well plates (1.5 × 10^5^/well). The monolayer was scratched to introduce a wound and treated with Con-CM or GDF15-CM. The migration of cells into the wound area was analyzed after 24 h (significance compared with the cells cultured in Con-CM). **(E)** Tube formation assay results. HBMVECs (1.5 × 10^5^ cells) were incubated on Matrigel matrix for 3 days with Con-CM or GDF15-CM. The tube length was measured using ImageJ angiogenesis analyzer (significance was compared with the cells cultured in Con-CM). (*p < 0.05 and ***p < 0.001 compared with the cells cultured in Con-CM).

**Figure 5:**
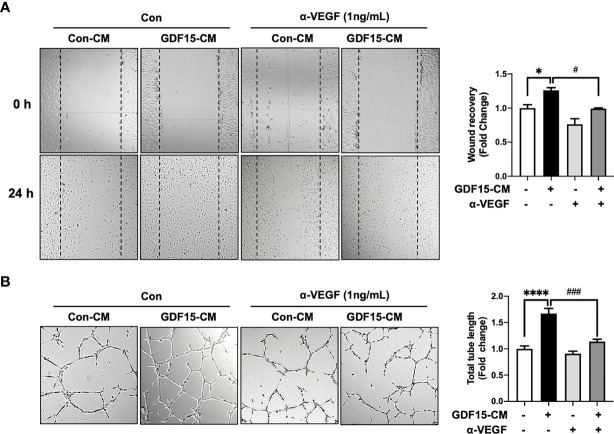
Anti-VEGF antibody blocks GDF15 mediated angiogenesis. **(A)** Wound healing assay was performed using human brain microvascular endothelial cells (HBMVECs) in the absence/presence of anti-VEGF antibody. The day before the experiment, HBMVECs were seeded in 12-well plates (1.5 × 10^5^/well). The monolayer was scratched to introduce a wound and cells were cultured with control conditioned medium (Con-CM; conditioned medium from HBMVECs) or GDF15-CM HBMVECs (conditioned medium from rhGDF15-stimulated HBMVECs) with added control antibody or α-VEGF (1 ng/mL each). The wounded areas were photographed at 0 h and 24 h and the cell migration as percent mean distance migration was assessed. **(B)** Representative photographs of tube formation assay. HBMVECs (1.5 × 10^5^ cells) were incubated on Matrigel matrix for 12 h with Con-CM or GDF15-CM in the presence or absence of α-VEGF (1 ng/mL). The tube length was measured using ImageJ angiogenesis analyzer (significance was compared with the group Con-CM in the absence of α-VEGF). Data are presented as the mean ± standard error (*p < 0.01 and ****p < 0.001 compared with the non-treated CM control; ^#^ *p* < 0.05 and ^###^ *p* < 0.001 compared with rhGDF15- CM group).

To investigate the role of GDF15 in the interaction between ECs and glioblastoma cells, GDF15-induced VEGFA expression in glioblastoma cells and its effects on ECs were examined. The effect of conditioned medium (CM) derived from rhGDF15-stimulated U373 cell cultures (GDF15-CM) on HBMVEC cultures was examined (**Figure 4A**). As GDF15-CM contains secreted VEGFA, KDR levels were examined in HBMVECs. GDF15-CM upregulated *KDR* mRNA levels and increased KDR phosphorylation in HBMVECs (**Figure 4B, C**). Next, HBMVECs cultured in GDF15-CM were subjected to wound healing and tube formation assays. The migration (2.3-fold; *p* < 0.05) and tube formation (1.2-fold; *p* < 0.001) abilities of HBMVECs cultured in GDF15-CM were significantly higher than those of HBMVECs cultured in Con-CM (control CM, culture media derived from U373 cells not treated with rhGDF15) (**Figure 4D, E**). Further, to validate GDF15-induced VEGF secretion, we performed would healing assay and tube formation assays using a neutralizing VEGF antibody (α-VEGF). We added neutralizing VEGF antibody (1 ng/mL) to Con-CM and GDF15-CM, which were incubated with HBMVECs for 24 h. By neutralizing VEGF, the migration (27%, *p* < 0.05) and tube formation (53%, *p* < 0.01) abilities of both cultures were notably blocked compared to those in the absence of α-VEGF (**Figure 5**).

### SiRNA-Mediated Knockdown of *GDF15* Suppresses Radiation-Induced HBMVEC Migration

**Figure 6:**
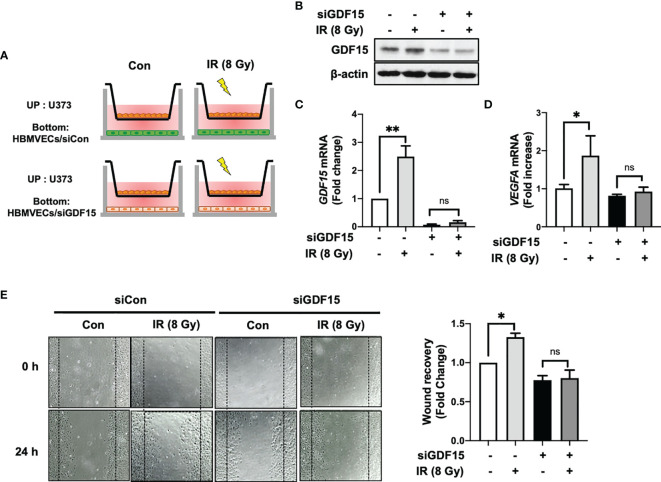
*GDF15* knockdown suppresses ionizing radiation (IR)-induced wound recovery. **(A)** Graphic experimental scheme; for the co-culture of U373 cells and human brain microvascular endothelial cells (HBMVECs), U373 cells were seeded on the upper well and the siGDF15-transfected or siCon-transfected HBMVECs were seeded on the bottom well. After irradiation with 8 Gy IR, U373 cells and HBMVECs were co-cultured for 24 h. **(B)** In HBMVECs, the protein levels of GDF15 were examined by immunoblotting analysis 24 h post-IR exposure. **(C)** In HBMVECs, the levels of *GDF15* mRNA were determined using quantitative reverse transcription polymerase chain reaction 24 h post-IR exposure. **(D)** In U373 cells, the levels of *VEGFA* mRNA were determined using qRT-PCR 24 h post-IR exposure. **(E)** For the wound healing assay, the HBMVECs on the plate were scratched and incubated for 24 h after irradiation with 8 Gy IR. The wound recovery rates were assessed using ImageJ. Data are presented as the mean ± standard deviation of three experiments. *p < 0.05 and **p < 0.01 compared with siCon-transfected and nonirradiated HBMVECs. ns, no significance.

In addition to ablating cancer cells, IR promotes angiogenesis. IR stimulates the angiogenic activity of ECs in the tumor (24, 25). To examine the involvement of GDF15 in IR-induced tumor angiogenesis through VEGFA regulation, the effect of *GDF15* knockdown on HBMVEC angiogenic activity was examined using the HBMVEC–U373 co-culture system (**Figure 6A**). We confirmed that siGDF15 treatment inhibited the protein levels of GDF15 in HBMVECs (**Figure 6B**). IR-induced GDF15 expression in siCon-transfected HBMVECs was upregulated 2.49-fold (*p* = 0.0027) compared with that in siGDF15-transfected HBMVECs. In contrast, IR did not affect GDF15 expression in siGDF15-transfected HBMVECs (**Figure 6C**). IR-induced VEGFA expression in U373 cells co-cultured with siCon-transfected HBMVECs (*p* = 0.0198) was 1.87-fold higher than that in U373 cells co-cultured with siGDF15-transfected HBMVECs. Irradiation with IR did not affect VEGF expression in U373 cells co-cultured with siGDF15-transfected HBMVECs (**Figure 6D**). To investigate the effects of *GDF15* knockdown on angiogenic activity mediated by the interaction between glioblastoma cells and ECs, the effect of IR on wound healing in siCon-transfected or siGDF15-transfected HBMVECs co-cultured with U373 cells was examined. Transfected HBMVECs in the lower well were scratched, irradiated with IR, and incubated with U373 cells for 24 h. Irradiation with IR did not affect the wound recovery rates in siGDF15-transfected HBMVECs (**Figure 6E**). In contrast, the wound recovery rates in siCon-transfected HBMVECs increased 1.33-fold compared to those in siGDF15-transfected HBMVECs (*p* = 0.0268).

### GDF15 Accelerates *In Vivo* Glioma Angiogenesis by Stimulating VEGFA Secretion

**Figure 7:**
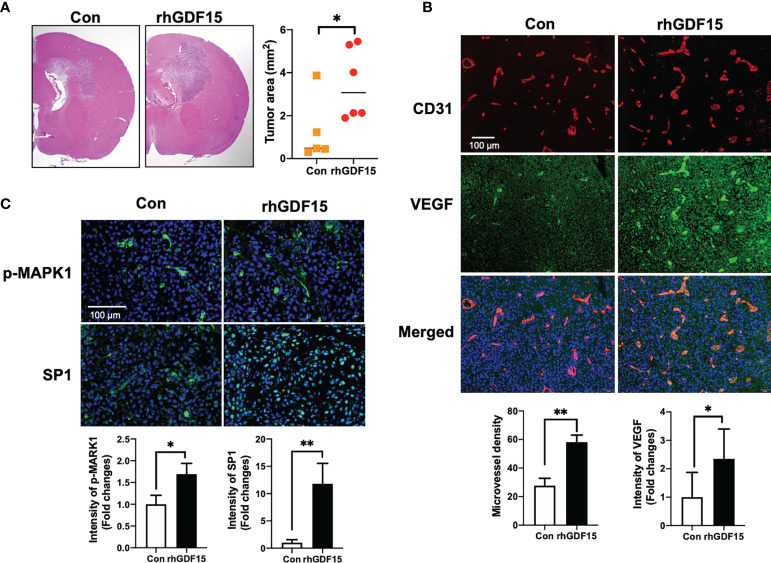
GDF15 promotes angiogenesis by stimulating VEGFA secretion in the brain tumor. **(A)** Representative section of the brain tumors from mice injected with control U373 cells and rhGDF15-stimulated U373 cells. The brain sections were stained with hematoxylin and eosin. The brain tumor area was measured using ImageJ. **(B)** VEGFA (green) and CD31-positive endothelial cells (red) in mouse brain tumor tissues were analyzed using immunofluorescence staining. Microvessel density was counted as the number of CD31-positive vessels. Scale bar = 100 µm. **(C)** Representative image of immunofluorescence analysis of the phosphorylation of MAPK1 (p-MAPK1; upper panel; green) and expression of SP1 (upper panel; green) counterstained with 4′,6-diamidino-2-phenylindole (DAPI) (blue). Scale bar = 100 µm. Fluorescence intensity was calculated using ImageJ as follows: Corrected total cell fluorescence = integrated density − (area of selected cell × mean fluorescence of background readings). Data are presented as the mean ± standard (control group, n = 5; rhGDF15-treated group; n = 6), * *p* < 0.05 and ** *p* < 0.01 compared with control group.

To investigate the effect of GDF15 on brain tumors *in vivo*, orthotropic brain tumors were injected with rhGDF15-stimulated U373 cells (1 × 10^5^ cells; cultured in the presence of 50 ng/mL rhGDF15 for 2 weeks). Mice with orthotropic brain tumors injected with control U373 cells (not treated with rhGDF15) served as a control group. The *VEGFA* mRNA expression levels in rhGDF15-stimulated U373 cells were confirmed using qRT-PCR before stereotactically injecting the cells into the brain tumors (**Supplementary Figure S3**). The mice were euthanized, and the tumor size was measured in the H-E-stained brain sections on day 10 post-injection. The tumor size was significantly higher in the rhGDF15-stimulated U373 cell-injected group than in the control U373 cell-injected group (**Figure 7A**). Immunofluorescence analysis of the tumor sections revealed that the EC density (represented by CD31-positive vessels) in the rhGDF15-stimulated U373 cell-injected tumors was significantly higher than that in the control U373 cell-injected tumors. VEGFA-positive signals in both cancer cells and ECs of the rhGDF15-stimulated U373 cell-injected tumors were significantly higher than those in both cancer cells and ECs of the control U373 cell-injected tumors (**Figure 7B**). We also performed immunofluorescence analysis of p-MAPK1 and SP1 levels in the control- and GDF15-treated tumors. Consistent with the *in vitro* results, the rhGDF15-treated U373 cell-injected tumors exhibited higher levels of both p-MAPK1 and SP1 than control tumors (**Figure 7C**). We also analyzed the expression *GDF15* and *VEGFA* mRNA in the tumors from the control and rhGDF15-treated groups using qRT-PCR. The mRNA expression of both *GDF15* and *VEGFA* was higher in the rhGDF15-treated group than in the control group (**Supplementary Figure S4**).

## Discussion

As angiogenesis is a key event for the progression of GBM, various studies have proposed chemotherapy, radiation, or combined treatment modalities to inhibit this process in GBM (3, 4). However, radiation can stimulate angiogenesis by promoting the release of various cytokines from different cell types, including cancer cells, immune cells, and ECs, into the glioma microenvironment (26, 27). To inhibit radiation-induced angiogenesis, the interactions between various components of the tumor microenvironment must be elucidated. Therefore, this study examined the role of GDF15, which is one of the cytokines released from the tumor microenvironment, in the cross-talk between ECs and glioma cells irradiated with IR. The findings of this study indicated that radiation-induced HBMVEC-derived GDF15 promoted VEGFA production in U373 glioma cells and consequently enhanced angiogenesis in glioma.

**Figure 8:**
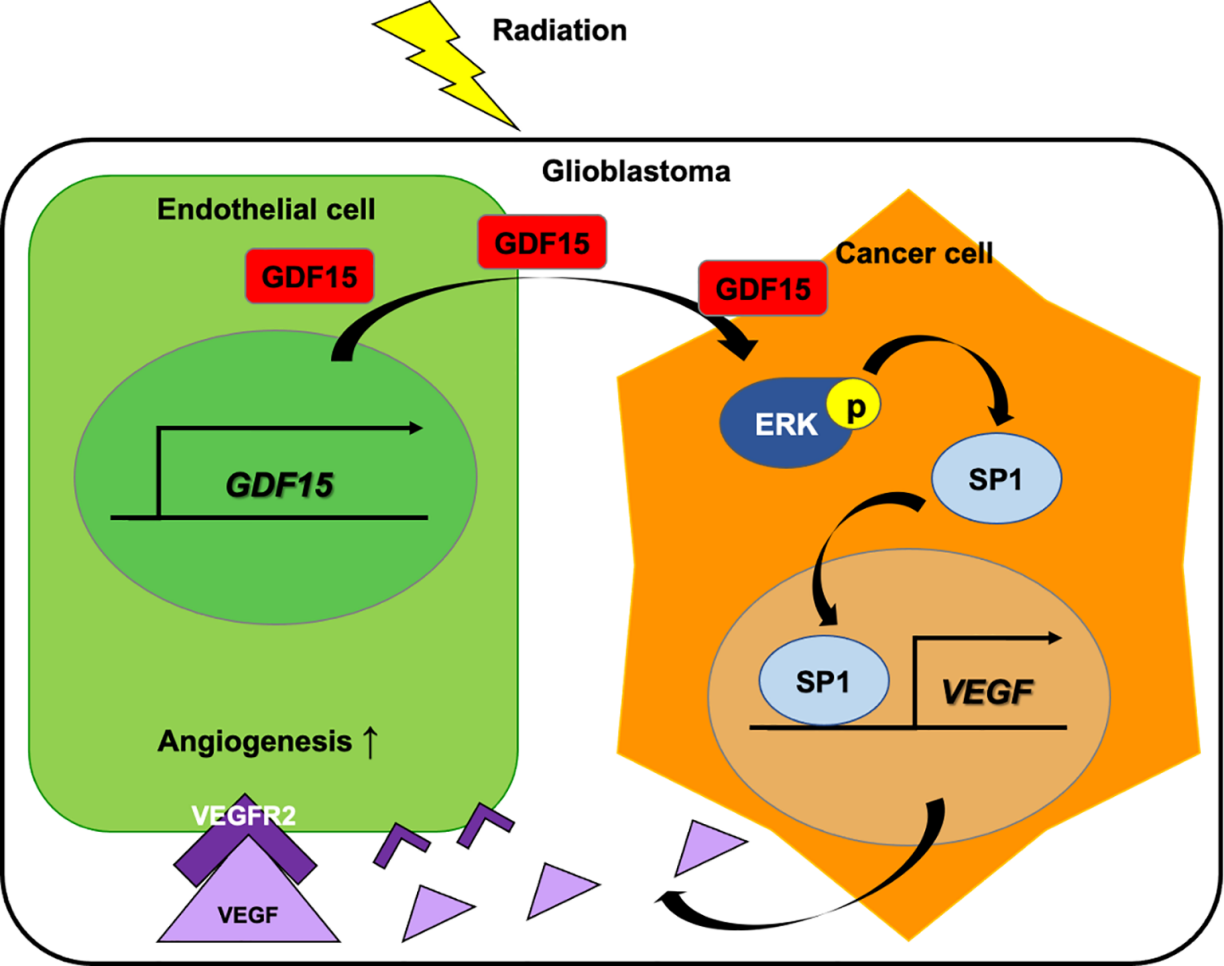
Role of GDF15 in ionizing radiation (IR)-induced angiogenesis in glioblastoma. Radiation upregulates GDF15 expression in the endothelial cells (ECs). EC-derived GDF15 activates the p-MAPK1/SP1 pathway in glioblastoma cells. SP1 stimulates the promoter activity of VEGF. The upregulated VEGF levels induce angiogenesis in glioblastoma through p-KDR on ECs.

In this study, IR directly upregulated GDF15 expression and secretion in HBMVECs (**Figure 1**). The upregulated GDF15 levels in the blood or cerebrospinal fluid are reported to be correlated with poor survival in patients with GBM (28). Hence, the effect of rhGDF15 on the proliferation and migration abilities of glioma cells was examined in this study. Ideally, the CM should be collected from the ECs following IR. However, radiation stimulates the release of diverse angiocrine factors from ECs post IR (29). Since the irradiated CM would contain other cytokines in addition to GDF15, we cannot be certain whether the induction of VEGF expression is caused by GDF15 alone or by other factors. Therefore, we determined whether GDF15 directly modulated proliferation/migration of glioma cells or induced VEGF expression in U373 cells (**Figure 2**). GDF15 is reported to promote the growth of ovarian and prostate cancers (16, 17). However, rhGDF15 treatment did not affect the proliferation and migration of U373 cells in this study but markedly promoted VEGFA production in U373 cells through the p-MAPK1/SP1 pathway. This finding is consistent with that of Griner et al. (16) who reported that exogenous GDF15 treatment and endogenous GDF15 overexpression stimulated the phosphorylation of p38, Erk1/2, and Akt. Consistent with the findings of this study, Mielcarska et al. reported that increased GDF15 levels were correlated with VEGFA production in colorectal cancer. These findings demonstrate the mechanism of GDF15 in radiation-induced angiogenesis in glioma. As shown in **Figure 8**, GDF15 promotes tumor angiogenesis through a positive feedback loop comprising EC-secreted GDF15, glioma-derived VEGFA, and KDR on ECs. IR promotes GDF15 secretion from the ECs, which leads to the secretion of VEGF from the glioma cells. VEGF activates ECs by binding to KDR and consequently promotes angiogenesis. To the best of our knowledge, this is the first study to propose a mechanism for GDF15-mediated angiogenesis involving the cross-talk between ECs and glioma cells.

Furthermore, this study demonstrated the *in vivo* effect of GDF15 on brain tumor angiogenesis. Consistent with the *in vitro* results, we observed that orthotopic brain tumors injected with rhGDF15-exposed glioma cells showed increased VEGF expression and enhanced angiogenesis than control tumors. However, this study has some limitations. First, to validate whether the observed angiogenesis in the tumors is mediated by an increase in VEGF expression levels in U373 cells, further *in vivo* studies using VEGF inhibiting agents or VEGF-knockdown U373 cells are needed. Second, this study only examined the interaction between ECs and glioma cells using rhGDF15. Generally, IR dose fractionation is administered to brain tumor patients over several weeks. In fact, brain tumor cells are continuously and repeatedly exposed to the GDF15-secreting ECs. We treated U373 cells with rhGDF15 for 2 weeks prior to injecting them into the mouse brains to mimic the secretion of GDF15 from ECs to the tumor microenvironment. Therefore, our method does not sufficiently mimic the actual tumor microenvironment *in vivo*. Nevertheless, we can reveal the association of tumor growth and angiogenesis with GDF15 alone. Finally, complex intercellular communication is involved in tumor progression and angiogenesis. Therefore, further studies are required to examine the interaction of GDF15 with other components of the tumor microenvironment, including macrophages and fibroblasts.

In summary, this study demonstrated that IR directly promotes the secretion of GDF15 from brain ECs and that GDF15 activates the *VEGFA* promoter in glioma cells through the p-MAPK1/SP1 pathway and consequently enhances angiogenesis in orthotopic brain tumors. These results suggest that radiotherapy-induced GDF15 secretion may contribute to tumor recurrence or therapy resistance by mediating the cross-talk between brain cancer cells and ECs. Therefore, GDF15 is a potential therapeutic target for glioblastoma.

## Data Availability Statement

The datasets presented in this study can be found in online repositories. The names of the repository/repositories and accession number(s) can be found in the article/**Supplementary Material**.

## Ethics Statement

The animal study was reviewed and approved by Institutional Animal Care and Use Committee of the Korea Institute of Radiological and Medical Sciences (Approval no. KIRAMS2018-0079). Written informed consent was obtained from the owners for the participation of their animals in this study.

## Author Contributions

HP and K-SN performed molecular and cellular experiments. HP generated and analyzed the data. HP and H-JL designed and performed animal experiments. H-JL and KK reviewed the data, prepared the manuscript, and supervised the work. All authors contributed to the article and approved the submitted version.

## Funding

This work was supported by grants from the National Research Foundation (NRF-2020M2C8A2069337) and a grant from the Korea Institute of Radiological and Medical Sciences (50531–2021), which is funded by the Ministry of Science and Information and Communications Technology of the Korean government.

## Conflict of Interest

The authors declare that the research was conducted in the absence of any commercial or financial relationships that could be construed as a potential conflict of interest.

## Publisher’s Note

All claims expressed in this article are solely those of the authors and do not necessarily represent those of their affiliated organizations, or those of the publisher, the editors and the reviewers. Any product that may be evaluated in this article, or claim that may be made by its manufacturer, is not guaranteed or endorsed by the publisher.

## Abbreviations

CM, Conditioned medium; ECs, Endothelial cells; GBM, Glioblastoma; HBMVECs, Human brain microvascular endothelial cells; H-E, Hematoxylin and Eosin; IR, Ionizing radiation; KDR, Vascular endothelial growth factor receptor 2; rhGDF15, Recombinant human GDF15; RT, Room temperature; VEGF, Vascular endothelial growth factor.

## References

[1] Bulnes S Bengoetxea H Ortuzar N Argandona EG Garcia-Blanco A Rico-Barrio I. Angiogenic Signalling Pathways Altered in Gliomas: Selection Mechanisms for More Aggressive Neoplastic Subpopulations With Invasive Phenotype. J Signal Transduct (2012) 2012:597915. doi: 10.1155/2012/597915 22852079 PMC3407647

[2] Plate KH Scholz A Dumont DJ. Tumor Angiogenesis and Anti-Angiogenic Therapy in Malignant Gliomas Revisited. Acta Neuropathol (2012) 124(6):763–75. doi: 10.1007/s00401-012-1066-5 PMC3508273 23143192

[3] Ahir BK Engelhard HH Lakka SS. Tumor Development and Angiogenesis in Adult Brain Tumor: Glioblastoma. Mol Neurobiol (2020) 57(5):2461–78. doi: 10.1007/s12035-020-01892-8 PMC7170819 32152825

[4] Wong ML Prawira A Kaye AH Hovens CM. Tumour Angiogenesis: Its Mechanism and Therapeutic Implications in Malignant Gliomas. J Clin Neurosci (2009) 16(9):1119–30. doi: 10.1016/j.jocn.2009.02.009 19556134

[5] Hida K Maishi N Annan DA Hida Y. Contribution of Tumor Endothelial Cells in Cancer Progression. Int J Mol Sci (2018) 19(5):1272. doi: 10.3390/ijms19051272 PMC5983794 29695087

[6] Zuazo-Gaztelu I Casanovas O. Unraveling the Role of Angiogenesis in Cancer Ecosystems. Front Oncol (2018) 8:248. doi: 10.3389/fonc.2018.00248 30013950 PMC6036108

[7] Tamura R Tanaka T Miyake K Yoshida K Sasaki H. Bevacizumab for Malignant Gliomas: Current Indications, Mechanisms of Action and Resistance, and Markers of Response. Brain Tumor Pathol (2017) 34(2):62–77. doi: 10.1007/s10014-017-0284-x 28386777

[8] Yock TI Tarbell NJ. Technology Insight: Proton Beam Radiotherapy for Treatment in Pediatric Brain Tumors. Nat Clin Pract Oncol (2004) 1(2):97–103. doi: 10.1038/ncponc0090 16264827

[9] Seo YS Ko IO Park H Jeong YJ Park JA Kim KS. Radiation-Induced Changes in Tumor Vessels and Microenvironment Contribute to Therapeutic Resistance in Glioblastoma. Front Oncol (2019) 9:1259. doi: 10.3389/fonc.2019.01259 31803626 PMC6873882

[10] Kim EJ Lee H Lee YJ Sonn JK Lim YB. Ionizing Radiation Regulates Vascular Endothelial Growth Factor-A Transcription in Cultured Human Vascular Endothelial Cells *Via* the PERK/eIF2alpha/ATF4 Pathway. Int J Radiat Oncol Biol Phys (2020) 107(3):563–70. doi: 10.1016/j.ijrobp.2020.03.003 32169411

[11] Moeller BJ Cao Y Li CY Dewhirst MW. Radiation Activates HIF-1 to Regulate Vascular Radiosensitivity in Tumors: Role of Reoxygenation, Free Radicals, and Stress Granules. Cancer Cell (2004) 5(5):429–41. doi: 10.1016/s1535-6108(04)00115-1 15144951

[12] Coll AP Chen M Taskar P Rimmington D Patel S Tadross JA. GDF15 Mediates the Effects of Metformin on Body Weight and Energy Balance. Nature (2020) 578(7795):444–8. doi: 10.1038/s41586-019-1911-y PMC7234839 31875646

[13] Ost M Igual Gil C Coleman V Keipert S Efstathiou S Vidic V. Muscle-Derived GDF15 Drives Diurnal Anorexia and Systemic Metabolic Remodeling During Mitochondrial Stress. EMBO Rep (2020) 21(3):e48804. doi: 10.15252/embr.201948804 32026535 PMC7054681

[14] Kempf T Eden M Strelau J Naguib M Willenbockel C Tongers J. The Transforming Growth Factor-Beta Superfamily Member Growth-Differentiation Factor-15 Protects the Heart From Ischemia/Reperfusion Injury. Circ Res (2006) 98(3):351–60. doi: 10.1161/01.RES.0000202805.73038.48 16397141

[15] Park H Kim CH Jeong JH Park M Kim KS. GDF15 Contributes to Radiation-Induced Senescence Through the ROS-Mediated P16 Pathway in Human Endothelial Cells. Oncotarget (2016) 7(9):9634–44. doi: 10.18632/oncotarget.7457 PMC4891072 26909594

[16] Griner SE Joshi JP Nahta R. Growth Differentiation Factor 15 Stimulates Rapamycin-Sensitive Ovarian Cancer Cell Growth and Invasion. Biochem Pharmacol (2013) 85(1):46–58. doi: 10.1016/j.bcp.2012.10.007 23085437 PMC4329765

[17] Wang W Yang X Dai J Lu Y Zhang J Keller ET. Prostate Cancer Promotes a Vicious Cycle of Bone Metastasis Progression Through Inducing Osteocytes to Secrete GDF15 That Stimulates Prostate Cancer Growth and Invasion. Oncogene (2019) 38(23):4540–59. doi: 10.1038/s41388-019-0736-3 PMC9097780 30755731

[18] Lu X He X Su J Wang J Liu X Xu K. EZH2-Mediated Epigenetic Suppression of GDF15 Predicts a Poor Prognosis and Regulates Cell Proliferation in Non-Small-Cell Lung Cancer. Mol Ther Nucleic Acids (2018) 12:309–18. doi: 10.1016/j.omtn.2018.05.016 PMC6031151 30195769

[19] Vanhara P Hampl A Kozubik A Soucek K. Growth/differentiation Factor-15: Prostate Cancer Suppressor or Promoter? Prostate Cancer Prostatic Dis (2012) 15(4):320–8. doi: 10.1038/pcan.2012.6 22370725

[20] Wang X Baek SJ Eling TE. The Diverse Roles of Nonsteroidal Anti-Inflammatory Drug Activated Gene (NAG-1/GDF15) in Cancer. Biochem Pharmacol (2013) 85(5):597–606. doi: 10.1016/j.bcp.2012.11.025 23220538 PMC3566326

[21] Ramanathan M Giladi A Leibovich SJ. Regulation of Vascular Endothelial Growth Factor Gene Expression in Murine Macrophages by Nitric Oxide and Hypoxia. Exp Biol Med (Maywood) (2003) 228(6):697–705. doi: 10.1177/153537020322800608 12773701

[22] Curry JM Eubank TD Roberts RD Wang Y Pore N Maity A. M-CSF Signals Through the MAPK/ERK Pathway *via* Sp1 to Induce VEGF Production and Induces Angiogenesis *In Vivo* . PloS One (2008) 3(10):e3405. doi: 10.1371/journal.pone.0003405 18852899 PMC2566603

[23] Feng J Zhang Y Xing D. Low-Power Laser Irradiation (LPLI) Promotes VEGF Expression and Vascular Endothelial Cell Proliferation Through the Activation of ERK/Sp1 Pathway. Cell Signal (2012) 24(6):1116–25. doi: 10.1016/j.cellsig.2012.01.013 22326662

[24] Yana I Sagara H Takaki S Takatsu K Nakamura K Nakao K. Crosstalk Between Neovessels and Mural Cells Directs the Site-Specific Expression of MT1-MMP to Endothelial Tip Cells. J Cell Sci (2007) 120(Pt 9):1607–14. doi: 10.1242/jcs.000679 17405818

[25] Bouquet F Pal A Pilones KA Demaria S Hann B Akhurst RJ. TGFbeta1 Inhibition Increases the Radiosensitivity of Breast Cancer Cells *In Vitro* and Promotes Tumor Control by Radiation *In Vivo* . Clin Cancer Res (2011) 17(21):6754–65. doi: 10.1158/1078-0432.CCR-11-0544 PMC3724539 22028490

[26] Tabatabai G Frank B Mohle R Weller M Wick W. Irradiation and Hypoxia Promote Homing of Haematopoietic Progenitor Cells Towards Gliomas by TGF-Beta-Dependent HIF-1alpha-Mediated Induction of CXCL12. Brain (2006) 129(Pt 9):2426–35. doi: 10.1093/brain/awl173 16835250

[27] Zhou W Jiang Z Li X Xu Y Shao Z. Cytokines: Shifting the Balance Between Glioma Cells and Tumor Microenvironment After Irradiation. J Cancer Res Clin Oncol (2015) 141(4):575–89. doi: 10.1007/s00432-014-1772-6 PMC11824017 25005789

[28] Codo P Weller M Kaulich K Schraivogel D Silginer M Reifenberger G. Control of Glioma Cell Migration and Invasiveness by GDF-15. Oncotarget (2016) 7(7):7732–46. doi: 10.18632/oncotarget.6816 PMC4884950 26741507

[29] Nolan DJ Ginsberg M Israely E Palikuqi B Poulos MG James D. Molecular Signatures of Tissue-Specific Microvascular Endothelial Cell Heterogeneity in Organ Maintenance and Regeneration. Dev Cell (2013) 26(2):204–19. doi: 10.1016/j.devcel.2013.06.017 PMC3873200 23871589

