# Supplementary material for: Ionizing Radiation-Induced GDF15 Promotes Angiogenesis in Human Glioblastoma Models by Promoting VEGFA Expression Through p-MAPK1/SP1 Signaling
Source: Front Oncol. 2022 Feb 25;12:801230. doi: 10.3389/fonc.2022.801230 (PMC8913883; doi:10.3389/fonc.2022.801230)
Supplement: Supplementary file 1 [file DataSheet_1.docx]

Supplementary Material

**Supplementary Figure S1**. The effect of GDF15 on human brain microvascular endothelial cells (HBMVEs). (A) Cell viability. HBMVECs were cultured with 100 ng/mL rhGDF15 protein in culture medium for 3 days, and the numbers of cells were counted. (B) Western blotting for MAPK1 and p-MAPK1. (C) Wound healing assay. The day before the experiment, HBMVECs were seeded in 12-well plates (1.5 × 10^5^/well). Then, the monolayer in each well was scratched with a 200 μL tip. The wounded areas were photographed at 0 h and at 24 h, and the cell migration as percent mean distance migration was assessed. (D) Tube formation assay. HBMVECs (1.5 × 10^5^ cells) were incubated on Matrigel matrix for 12 h in the presence or absence of rhGDF15 (100 ng/mL). The tube length was measured using ImageJ angiogenesis analyzer


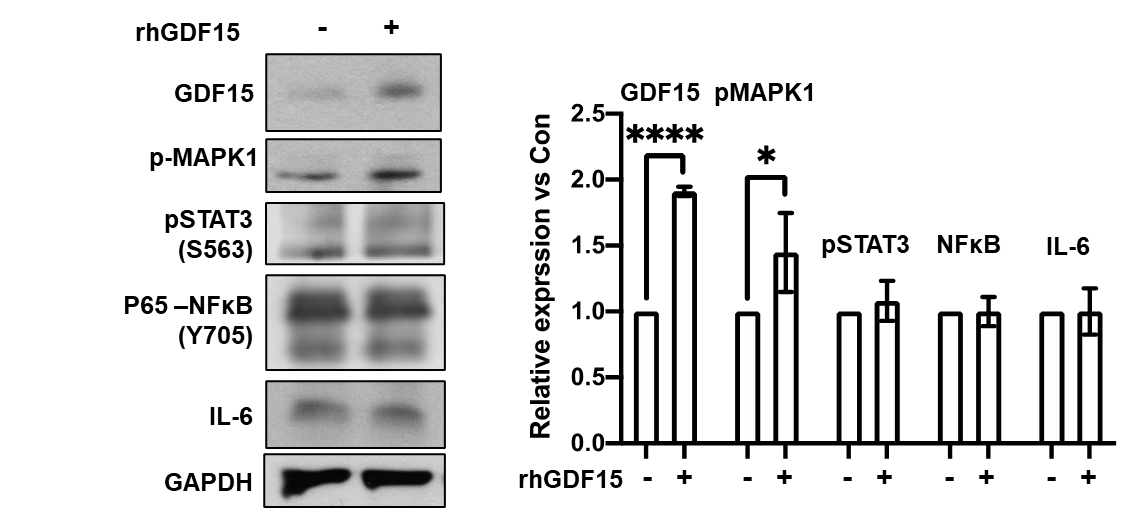


**Supplementary Figure S2.** Representative images of immunoblotting for GDF15, pMAPK1, pSTAT3, NF-κB, and IL-6. U373 cells were treated with rhGDF15 for 6 h and harvested for western blotting. Data are presented as the mean ± standard deviation (**p* < 0.05 and *****p* < 0.0001 compared with control group).

**Supplementary Figure S3**. *VEGF* mRNA levels in U373 cells. Prior to injecting U373 cells into the mouse brains, the cells were cultured with rhGDF15 (50 ng/mL culture medium) for 14 days. Data are presented as the mean ± standard (n = 3), * *p* < 0.05 compared with control U373 cells.

**Supplementary Figure S4**. mRNA levels of *GDF15* and *VEGFA* in the tumors. *GDF15* and *VEGFA* mRNA expression was analyzed by qRT-PCR in the tumors from the contro1 and rhGDF15-treated groups. Data are presented as the mean ± standard deviation (n = 3), * *p* < 0.05 compared with control group.

.
